# Exploring the barriers and enablers of diabetes care in a remote Australian context: A qualitative study

**DOI:** 10.1371/journal.pone.0286517

**Published:** 2023-07-27

**Authors:** Siobhan Bourke, Syarifah Liza Munira, Anne Parkinson, Emily Lancsar, Jane Desborough

**Affiliations:** Department of Health Services Research and Policy, National Centre for Epidemiology and Population Health, Australian National University, Canberra, Australia; Caleb University, NIGERIA

## Abstract

**Objective:**

This qualitative study explored the current barriers and enablers of diabetes care in the Indian Ocean Territories (IOT).

**Methods:**

A constructivist grounded theory approach that incorporated semi-structured telephone interviews was employed. Initial analysis of the interview transcripts used a line-by-line approach, to identify recurring themes, connections, and patterns, before they were re-labelled and categorised. This was followed by axial coding, categorisation refinement, and mapping of diabetes triggers in the IOT.

**Participants and setting:**

The IOT, consisting of Christmas Island and the Cocos (Keeling) Islands, are some of the most remote areas in Australia. When compared with mainland Australia, the prevalence of type 2 diabetes in the IOT is disproportionately higher. There were no known cases of type 1 diabetes at the time of the study. Like other remote communities, these communities experience difficulties in accessing health services to prevent and manage diabetes. Twenty health care professionals and health service administrators in the IOT took part in semi-structured telephone interviews held during April-June 2020. Participants included GPs, nurses, dietitians, social and community services workers, school principals, and administrators. The interview questions focused on their perceptions of the current diabetes care in place in the IOT and their views on the challenges of providing diabetes care in the IOT.

**Results:**

We identified four main barriers and two main enabling factors to the provision of effective diabetes care in the IOT. The barriers were: (i) societal influences; (ii) family; (iii) changing availability of food; (v) sustainability and communication. The two main enablers were: (i) tailoring interventions to meet local and cultural needs and values; and (ii) proactive compliance with the medical model of care.

**Conclusion:**

Due to the cultural and linguistic diversity within the IOT, many of the identified barriers and enablers are unique to this community and need to be considered and incorporated into routine diabetes care to ensure successful and effective delivery of services in a remote context.

## Introduction

Diabetes represents a major challenge to the Australian health system [1]. Type 1 diabetes (T1D) is less common than type 2 diabetes (T2D), which accounts for nearly 90% of cases globally and is recognised as one of the fastest growing chronic diseases worldwide [2, 3]. Individuals with T2D experience poor health outcomes, including increased risk of cardiovascular disease, kidney failure and diabetes-related eye problems. The management of T2D is complex as it requires significant long-term lifestyle changes, including improved diet, increased physical activity, better medication adherence, and self-management.

These lifestyle changes can be difficult to maintain and can be further complicated by social and economic factors, especially in rural and remote geographical areas, creating barriers to effective diabetes management. For example, in rural and remote areas, there is often poor and unreliable access to fresh foods and appropriate health services, resulting in detrimental health outcomes [4–6]. This is a point of concern in Australia, where the prevalence of T2D is significantly higher in rural and remote areas than in metropolitan areas [1]. This study explores the barriers and enablers of diabetes care in a remote Australian context—the Indian Ocean Territories (IOT), which are located 2700 kilometres (km) from Perth, Australia. As there were no known cases of type 1 diabetes in the IOT at the time of the study the focus is on type 2 diabetes care although the findings have relevance for both T1D and T2D care. Gestational diabetes was not within the scope of this study.

The IOT comprises two small isolated remote communities in Australia: Christmas Island and the Cocos (Keeling) Islands, which are about 900 km apart. Of the 27 Cocos (Keeling) Islands, only two small coral atolls are inhabited (Home Island and West Island) [7]. Population estimates suggest approximately 3000 people live on Christmas Island and about 545 people live on the Cocos (Keeling) Islands [8]. The IOT have some of the most culturally and linguistically diverse communities in Australia. On Christmas Island, more than half (50.9%) of households speak a non-English language [9], and on the Cocos (Keeling) Islands, it is nearly two-thirds (63.6%) of households [10]. Being external territories, the IOT islands are administered by Australia’s Department of Infrastructure, Transport, Regional Development and Communications (DITRDC). The IOT Health Service, a stand-alone health service managed by the DITRDC, provides inpatient, outpatient, and community-based health and dental services in the IOT. There is one clinic on Christmas Island comprising three GPs and 12 nurses and two clinics on the Cocos (Keeling) Islands comprising one GP and four nurses [11]. Most health professionals live in the IOT but some are based on mainland Australia and visit the IOT regularly. Complex and specialist procedures, emergency medicine, and birthing services, are provided in Western Australia (WA) to IOT residents through a service delivery arrangement between the DITRDC and the WA Government [11]. Any patients with gestational diabetes are managed by a child health antenatal nurse.

Prevalence rates for type 2 diabetes are known to increase with increasing remoteness area and are 1.3 and 1.4 times as high in remote and very remote areas, compared with major cities and inner regional areas [1]. For example, compared with the rate of type 2 diabetes in metropolitan Australia (3.8%) and in other remote areas in Australia (5.1%) [1], estimations suggest that there is a disproportionately higher rate of type 2 diabetes on Christmas Island (8%) and on the Cocos (Keeling) Islands (11%), respectively (8). A caveat here is that prevalence rates for the Australian population are likely to *underestimate* the true prevalence of T2D in the Australian population because they are based on people who have received a formal medical diagnosis of diabetes while many people are living with undiagnosed or pre-T2D [1]. This suggests the same may be true for those living in the IOT.

A number of studies have explored barriers and enablers to diabetes care for people with T2D living in a remote area [12–16]. A multinational study of diabetes self-management for people with either T1D or T2D [17] reported financial constraints, unrealistic expectations, and lack of advice about self-management as barriers. Enablers reported in the study included a determination to prevent diabetes complications and the use of health technology such as smart phone apps to assist self-management. Barriers and enablers of diabetes care have also been found to differ depending on geographical remoteness. For example, in remote or rural areas, reduced availability of fresh foods or limited specialised health care services were found to act as major barriers to effective care for people with T2D [14]. Other barriers identified in the literature include challenges with resource availability, staffing (retention and relationship building), and a reliance on reactive medicine rather than chronic care management [12–15].

Collectively, these identified barriers and enablers of diabetes care are important, but they may or may not be relevant to care for people with diabetes in the IOT. While the IOT is classified as remote Australia, they have a unique health service arrangement and a culturally and linguistically diverse population.

This study aimed to explore the current barriers and enablers of preventing and managing diabetes for people in the IOT from the perspective of those providing the care within the IOT Health Service, community leaders (for example, school principals), and administrators. Findings from this study will inform future policy interventions for diabetes care for people with T2D in the IOT where increasing presentations of people with T2D was identified as a key concern by the DITRDC, and contribute to our broader understanding of how to improve overall diabetes care in remote areas.

## Methods

### Design

We adopted a constructivist grounded theory analysis approach, which involves a process of iterative data collection and constant comparative analysis of the raw data, the literature, and the research memo to help inform the research; ensuring that the analysis and findings are “grounded” in participants’ own words and experiences [18–20]. The premise behind constructivist grounded theory is that “data do not provide a window on reality. Rather, the ‘discovered’ reality arises from the interactive process and its temporal, cultural, and structural contexts” [18]. This approach is well-suited to investigate dynamic and complex public health challenges composed of distinctive yet interrelated issues that together form a complete picture of systemic issues in a community [21]. Methods and results are reported according to the COREQ checklist [22].

### Data collection and recruitment

We employed purposive sampling for this study to ensure a mix of views drawn from a small population. Key stakeholders were identified as those employed by either the IOT Health Service, Department of Education, or DITRDC, and in a position to have input into the decision-making and running of the IOT Health Service, or provide clinical care, or provide student education to the IOT community. Consumers were not included at this time, and it was proposed to conduct a separate study at a later date to gain their insights.

Twenty-one individuals were invited (prior to the advent of the COVID-19 pandemic) to participate in one-on-one face-to-face interviews. They were based on either Christmas Island, the Cocos Keeling Islands, or mainland Australia. One health professional declined to participate (no reason was provided). Participants were recruited by the Manager of the IOT Health Service and included GPs, nurses, social and community workers, dietitians, allied health workers and administration staff. In addition, two IOT school principals were invited due to their strong connection to the community. Participants provided verbal consent to take part in the study at the beginning of each interview and were asked to confirm that they had read and understood the information sheet provided, had had any questions answered to their satisfaction, gave permission to be recorded and were happy to proceed. If a participant indicated they had not read the information sheet, it was read out to them by the interviewer.

Interviews were conducted by members of the research team (SB, LM, and EL) between April and June 2020. While initially planned to be conducted face -to-face, the COVID-19 pandemic disrupted travel and we were forced to conduct the interviews by telephone. The interviewers reflected this was more challenging. We had planned to meet participants at their various workplaces to talk through the study and build a relationship before any interviews took place, but this was not possible. Also, due to issues of connectivity common in remote locations, interviews online using Zoom or Microsoft Teams was not an option, and even the phone lines were not stable and often required calling back after calls dropped out, however, participants were very accommodating and recognised the impact of the pandemic and the need to adapt. Two team members (AP, JD) were experienced qualitative researchers and coached those conducting the interviews, debriefing them after their initial interview, and mentoring them throughout. The team comprised people with experience working with culturally and linguistically diverse communities and who were cognisant of being sensitive to participants’ cultural values and treating them with respect.

Interviews lasted on average one hour and were audio recorded and professionally transcribed verbatim with any identifying information removed to preserve confidentiality, although in a small community this cannot be guaranteed. Data saturation was reached after 20 interviews, with no new information arising.

### Interview guide

A semi-structured interview guide was developed by SB, LM, and EL (S1–S3 Tables) and was reviewed for flow and clarity of the questions by AP and JD. Interview questions focused on the topic of diabetes care for people with T2D in the IOT; more specifically, current practices, and barriers and enablers of T2D care in the IOT. General probing questions were used during the interviews to further clarify and discuss the topic in depth. After eight interviews were conducted, data collection was paused to conduct a preliminary analysis based on the researchers’ memos. The preliminary analysis reflected on the interview experience and considered content collected to that point. As a result, additional questions were added to gain further understanding of the community and their reactions to diabetes, obesity and lifestyle impacts on individuals in the IOT. Disruption to care due to the emerging issue of COVID-19 was also considered.

### Data analysis

We employed constant comparative analysis throughout data collection to explore and identify concepts. Transcripts were uploaded to NVivo software for analysis [23]. Two researchers (LM, and SB) conducted the initial analysis of the interview transcripts using a line-by-line approach, to identify codes, connections, and patterns, before they were re-labelled and categorised. The wider research team (JD, AP, JA) met on two occasions to conduct axial coding, refining the categorisation and mapping of triggers of diabetes in the IOT.

**Fig 1:**
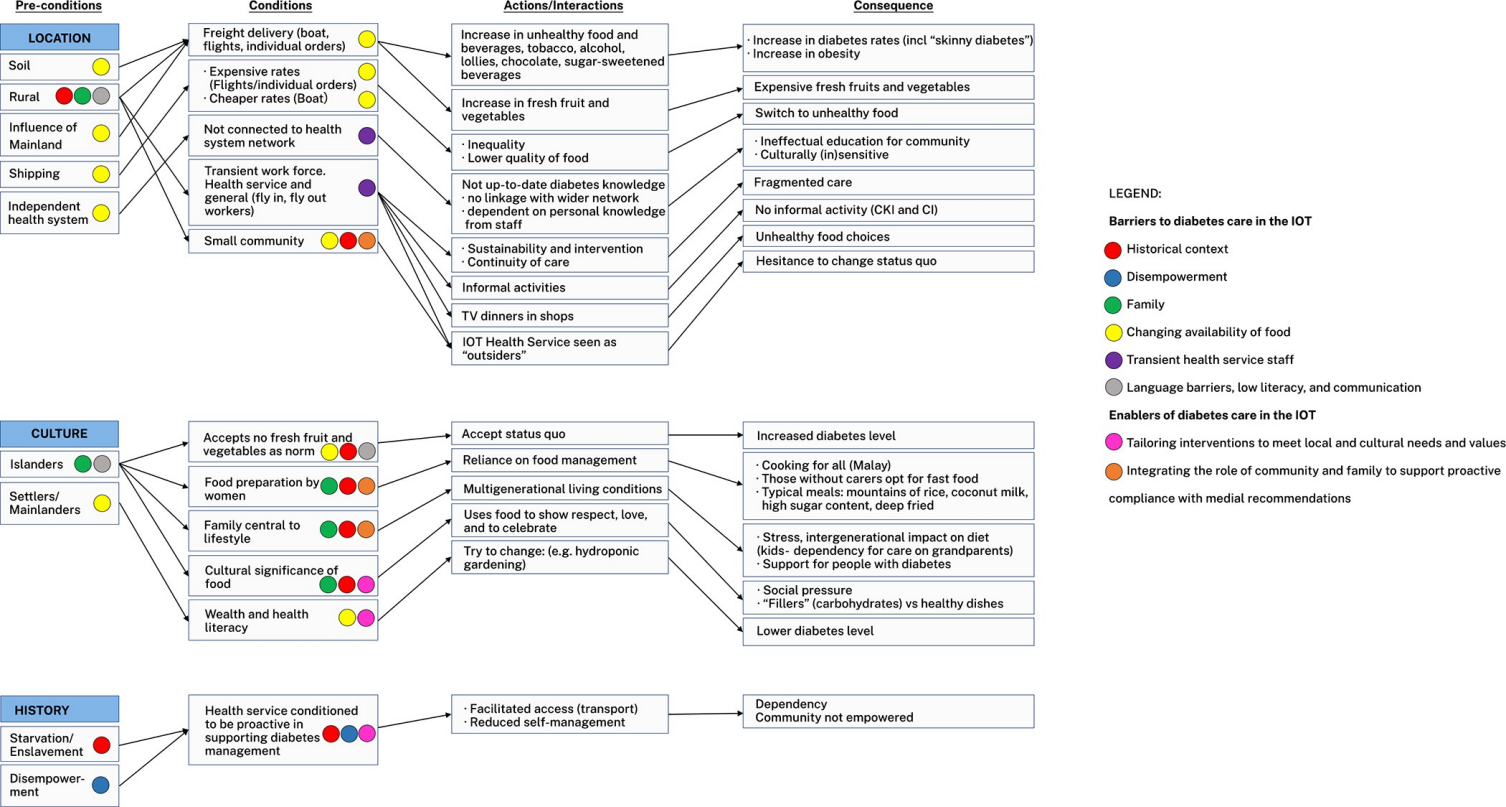
Path diagram describing participants’ perceptions of conditions, and actions and interactions underpinning diabetes care in the IOT, and related outcomes.

We compared responses across Christmas Island and the Cocos (Keeling) Islands to account for variability and credibility of the results. This approach facilitated the interpretation of how specific conditions supported actions and interactions, and participants’ perceived outcomes of this process [24]. A theoretical framework of barriers and enablers to diabetes care in the IOT was created by using a path diagram (Fig 1) [14]. Barriers and enablers were analysed as an outcome of a particular structure or process to explain the impact and consequences of these on diabetes care in the IOT. The researchers refined their thinking through an iterative process of reviewing the data and group discussion.

### Rigour

To ensure rigour, transcripts were analysed by two researchers (SB and LM) with authenticity achieved through verbatim transcription by a professional service and confirmation of this by listening to recordings. Researchers read the transcripts multiple times to ensure data familiarity. The researchers met daily during the data collection period, and afterwards met weekly to discuss and refine new concepts. Credibility of the data was established through ascertaining saturation. Identified patterns were refined as new data was collected. Validity of the findings were enhanced by incorporating findings from debriefings into the subsequent interviews and into the analysis. Feedback from the wider research team was incorporated to establish credibility. Quotes from participants with a range of views further supported accurate interpretation and rigour.

### Ethical considerations

Ethical approval was obtained from the Australian National University Human Research Ethics Committee (HREC Protocol 2019/687). Participants were asked to provide their informed (verbal) consent prior to their participation in the study, which was recorded and transcribed. Verbal consent was chosen to ensure participants understood the information sheet as it provided an opportunity for the interviewer to read out the information sheet and address any questions in real time. All participants were informed about how their anonymity and confidentiality would be managed in this study and advised that it could not be guaranteed due to the nature of a small community. Identifiable information from the interviews were removed, and the data (quotes) were tagged with participants’ location. As it is difficult to protect the identity of participants from a small community, we chose not to identify which Island they came from (Christmas Island or the Cocos (Keeling) Islands) rather we have identified them as being from either the IOT or mainland Australia.

## Results

In total, twenty participants were interviewed; 70% were female. Nine participants were based on the Cocos (Keeling) Islands, a further nine on Christmas Island, and two were based on the Australian mainland. The average length of time participants had resided in the IOT was 14 years (range = 3 months to 50 years) and the average length of experience working in the IOT Health Service was 8 years (range = 3 months to 43 years). Table 1 summarises participant characteristics.

**Table 1:** Participant characteristics: Health care professionals and health service administrators in the IOT.

Variable	No.
Participants (total)	20
Participants based on Cocos Keeling Islands	9
Participants based on Christmas Island	9
Participants based on mainland Australia	2
Female participants	6
Male participants	14
Average number of years residing in the Indian Ocean Territories (IOT)^a^	14.1 years
Average length of experience working in the IOT Health Service or Department of Infrastructure, Transport, Regional Development and Communications (DITRDC) ^b^	8.2 years

^a^ Data available for 17/20 participants only

^b^ Data available for 19/20 participants only

Participants were asked to reflect on their experiences of providing diabetes care for people with T2D and described a complex set of issues impacting T2D care in the IOT, highlighting both barriers and enablers. They described key factors that influenced dietary patterns, including economic, social and historical factors contributing to food availability and choices, and subsequently, islander health.

### Barriers to diabetes care in the IOT

We identified four main barriers to effective care for people with T2D in the IOT: (i) Societal influences; (ii) Family; (iii) Changing availability of food; (v) Sustainability and communication.

### Societal influences

#### Historical context and culture

A number of participants who had lived in the IOT for some time believed many of the factors that contribute to (un)healthy living in the IOT are largely reflective of the historical context of slavery and the geographic isolation of the islands, which has fostered a deep-seated connection between food insecurity and freedom.

*“…if we look at the island history of the fact that those people were stolen, enslaved, brought over there, imprisoned against their will from everything and everybody that they knew, and were basically reliant on these new masters for food. So, there was [a] scarce supply of food and definitely of quality. So, there are people on the island that still remember being hungry and starving… for them, food is a sign of success and freedom, and also part of their culture.”—IOT013*

The intergenerational impact of this trauma was repeatedly described by participants as having an impact on the consumption of (un)healthy food with a child’s foundation for a healthy lifestyle being shaped through the actions and habits of their household and extended family.

“*… the grandparents*, *they will go and buy their kids a chocolate bar*, *because they see that as a treat*, *because they’re thinking back to when they were their age*, *when they literally didn’t have enough food*. *They were starving and they were slaves*. *So*, *by buying a chocolate bar*, *that’s showing love and commitment to your grandchild…*. *It’s really*, *food is everywhere*, *food is everything*. *It’s a way of caring”—IOT020*

Participants perceived food as being attached to social status within the community. Food was seen to facilitate social interaction on the islands with eating and providing food perceived to be an act of caring and respect; bringing people together as a community. They also described the cultural custom of reciprocating respect, love and caring through food with large quantities of food linked to generosity and high social standing in the community.

“*I think you have women who their central way of socialising is surrounded around food*. *It’s also the way that they show*, *not who’s the best in the community*, *but show how much they love is by the more food that they have to offer*, *and the more food that they can bring to an event”—IOT018*

The cultural aspect of food sharing in the community was also related to increased portion sizes (using high carbohydrate “fillers”), which was observed by health staff and highlighted as a concern. These “filler” foods were used to bulk up serves to reduce costs, and in response to the lack of access to healthier food alternatives.

#### Disempowerment

Many participants recognised a tangible sense of disempowerment among the IOT community due to the impact of historical events which have resulted in deep-rooted and interwoven issues around empowerment and self-efficacy.

“*People weren’t encouraged*, *in fact they were actively discouraged to have any …self-actualisation*, *or self-efficacy*, *because it would go against the grain… It would go against the prescribed way things were supposed to be“—IOT018*

Historically, there was slow adoption of self-efficacy by those in the IOT. One participant described their experiences as a health worker residing in the IOT over a long period of time:

“*So self-management [of a chronic condition such as diabetes] is really*, *really new*. *Like when I went back in 1990*… *we had to get up*, *it was expected for us to get up at 2 o’clock in the morning to go and give people tablets because they couldn’t be trusted to take those tablets themselves at 2 o’clock in the morning*. *So*, *it’s come a huge way but I don’t think it’s got over that real hump yet of self-efficacy and self-management*, *and feeling*, *yeah*, *I can do this*. *It’s coming*, *but I don’t think it’s there yet”–IOT018*

This participant believed that slavery had significantly impacted the IOT population, creating a disempowered community reliant on direction by health professionals and lacking confidence to act on their own.

“*People do prefer*… *it’s a real*… *it’s so confusing because people like being told what to do*, *whereas in our*, *I suppose in present day people*, *like a lot of people in the community enjoy being told what to do*, *whereas it goes against the grain of [the] current medical approach*.*”—IOT018*

This sense of disempowerment has led to people in the community with T2D adopting the belief that good diabetes care equates to compliance and medical management of their diabetes rather than to making any lifestyle or behavioural changes.

Attendance at clinics for T2D management was high as medical management was valued but all the health professionals interviewed were frustrated that there was an ongoing lack of understanding about diabetes as a chronic condition.

“*Some people*, *when they are already on medication*, *they think they are fine*, *but they are not*. *We try to make sure their blood sugar is not too high*, *but they think being on tablets*, *they won’t have diabetes anymore*. *We try to explain again and again…”—IOT012*

Most health professionals believed there would be greater impact if patients could be encouraged to have more ownership over their own health and diabetes management.

“*It is very difficult when we explain to them at the clinic what is good and bad and they say “yes*, *yes*, *yes”*, *but when they go back*, *it’s a different story*. *I always say to them “this is not my body”… we try to encourage them to ring us; to come to the clinic*. *It’s in their interest to come and talk to us and ask questions*. *When we call them and ask if they have any questions they say “no*, *you called me”*.*”—IOT012*

### Family

Food preparation and management is a deeply rooted communal activity within households and communities. All participants identified that women in the IOT occupied a very traditional role in the home, especially in food preparation and management. The woman’s role was identified as a key target for any change, as their role could help drive the selection of healthy food choices and learning to prepare healthy foods.

“*…you can forget talking about healthy meal prep to the men*, *because they don’t do any cooking*, *so you’ve just got to focus on the women*. *You can talk to the men about snacks and soft drinks and that sort of stuff*. *But with the meals and that*, *you know*, *if … often the woman”—IOT019*

In the IOT communities, family structures frequently consisted of three or more generations based in the same household. Dinner and eating meals was often described as a ritual where island members regularly shared meals with their extended family. This presence of extended family members made the preparation of food complicated as they had to satisfy multiple preferences, making the transition to healthy foods for management of diabetes difficult. IOT Health Service staff found that in large families, food was prepared to cater for the majority of the family rather than for the person with diabetes.

“..*she won’t want to cook up four different types of meals for her household just because certain people have preferences about their diabetes*, *if that makes sense*. *You know*, *she might cook up a healthy meal*, *but then if half the household kicks up a stink*, *it’s no good*, *she’s just going to cook to the lowest common denominator in the household*.*”—IOT019*

A number of participants observed the difficulty faced by people living on their own, especially those without the support of family, where it seemed there was a lack of understanding about the importance of diet in managing T2D and little effort was made to prepare healthy food.

“*Some people have got a daughter at home who does the cooking and…could look after them and kind of maintain their diet*. *But some people don’t have that here*. *Some people just cook whatever they want*. *“—IOT014*.

### Changing availability of food

The lack of availability of affordable and healthy food was recognised by many participants as being a key influence on dietary habits. An increase in the volume of freight to the islands after the IOT’s integration with Australia in the mid-1980s, has resulted in less healthy and more highly processed foods arriving from the mainland. Some participants recognised traditional foods as being healthier than most of the food arriving from mainland Australia.

“*Before I was born*, *and probably there was no case of diabetes here because there were no soft drinks*, *people were like… if you speak to this other guy that’s been here longer than me and he’s been working in the department for long*, *like he said that there was no people*, *there’s not much case on diabetes until we became part of Australia when things starting to come in*, *all these drinks*, *all these chips*.*”—IOT012*

The influx and increased availability of unhealthy food coupled with the link between food and freedom has had a significant influence on food choices despite high food costs, thereby making lifestyle change difficult.

“*…it doesn’t help that everything’s expensive*, *but if it was solely a financial issue that dictated what people eat out here*, *they wouldn’t be spending money on all these additional snacks*, *treats*, *lollies*, *and soft drinks*.*”–IOT019*

### Sustainability and communication

#### Transient health workforce

When discussing current and past interventions for T2D implemented by the IOT Health Service, the issue of a transient workforce was repeatedly highlighted by many participants as a reason why these interventions were unsustainable.

“*…she [nurse] used to do like healthy cooking or organise different companies to come in and do like a check- up*, *check the BP [Blood Pressure]*. *And we used to have that*, *but she’s left… she’s not here anymore*. *But she used to have*, *you know*, *things like that happening here”—IOT014*

While many participants did see themselves as being part of the IOT community and highlighted the need for health services to display community values, there were other long-standing residents who believed that community members were skeptical of the health service professionals and their advice.

“*I don’t know*, *it’s very*… *or maybe I’m just too much in the forest to see the trees*, *you know*, *because people*, *you know it’s amazing*, *I hear these nurses saying*, *oh*, *you know*, *and this is so popular on Cocos Island*, *and this is so popular*, *but being in the community you sort of go*, *oh yeah*, *that’s what you think*, *but not really… So we’ll keep you happy*, *we’ll pop along*, *listen to what you say*, *but yeah*. *I mean look*, *I live in the community*, *and people come up to me and they say*, *oh I shouldn’t be eating this should I*? *And I’m like*, *it’s up to you*, *but there are probably better choices*, *but you know that’s up to you*. *And so they know*, *and they tell me*, *and then they eat it*.*”—IOT018*

This participant reinforced the importance of listening to the people on the ground providing services within the community and take on board their experiences to gain a sense of how the IOT Health Service is seen in reality by community members.

#### Language barriers, low literacy, and communication

All participants raised issues around language barriers and literacy and shared their experiences of trying to overcome these communication barriers and engage with people in their community. It was more evident on the Cocos (Keeling) Islands, particularly for the older population, where English is their second language.

“*But with the older ones*, *there’s definitely the communication barriers*, *i*.*e*. *language and translation*, *even trying to get the family to translate or a translator to translate*, *I don’t think it comes across the same*.*”—IOT017*

Clear communication between health professionals and people with T2D was often impacted by a reliance on family members to interpret or translate information into the primary language of a patient, hindering the sharing of important information about managing their T2D and treatment options. All participants from the IOT Health Service identified the language barrier as a key area to address and the need to increase availability of multi-language materials for the community. However, the issue of low literacy rates within the community, especially among older people, must also be overcome if health information is to be understood and acted upon.

“*Yeah*, *they don’t read*. *Some people don’t read*. *Some don’t know how to read*, *even though you write it in their language*, *yeah*, *they don’t read English*, *they don’t read Malay*, *they don’t do that”…The education is always there*, *every consult we give…*.*afterwards*, *they’ll just do whatever they like outside the clinic*, *you know…*.*there’s not*, *there can’t (*?*) be someone there to kind of maintain it*, *especially with the older people…with the younger generation they know how to maintain it*, *how to manage it*. *But for the older*, *you know*, *it’s kind of hard*. *They don’t know what they’re buying at the shops because they can’t read and*, *you know*, *they don’t even know… you know*, *even though you educate them*, *telling them this has lots of sugar*, *but*, *you know*, *they go to the shops*, *they can’t read*. *They’ll just buy anything they want*. *Yeah*. *This is with the older generation… the older patients that we have*.*”—IOT014*

Drawing on their experiences, some participants saw increasing the responsibility given to families of patients to synthesise and communicate complex health information as a positive action that could further increase the shared responsibility within the family unit to encourage and support self-care behaviour and management. Especially for the older population who were identified as being most resistant to making lifestyle changes, thereby posing a major barrier to T2D management despite excellent compliance with medication regimens and attending medical appointments. Although there was a perception among healthcare workers of hesitancy or resistance among patients to attend appointments for dietary consultations, which may also have been influenced by the dietitian not being based on IOT:

“*We’ve got the dietitian as well that’s been coming in [from mainland Australia]*. *But*, *you know*, *they don’t want to see her”—IOT019*

This highlights the difficulties of instigating sustained lifestyle behavioural changes and a deep-seated reluctance to change food habits.

### Enablers of diabetes care in the IOT

Two main enablers to effective T2D care in the IOT were identified from participant interviews: (i) tailoring interventions to meet local and cultural needs and values; and (ii) proactive compliance with the medical model of care.

### Tailoring interventions to meet local and cultural needs and values

Most participants agreed that more education regarding healthy eating was required for people with T2D. Although, in order to be effective, they reinforced the importance of it being tailored to the personal circumstances of each person, including being culturally appropriate and matched to their level of literacy:

“*We would–depending on the patient’s education*, *language backgrounds–my pitch to them will vary considerably*. *May or may not include also getting a Telehealth review with the dietitian [based in mainland Australia]*.*”—IOT019*

Many participants also explained that another challenge was that education provided by the IOT Health Service focused on western concepts of nutrition (e.g., increasing consumption of fresh fruits and vegetables), which was difficult for islanders to comprehend and implement due to structural and social barriers:

“*I think*, *the fact that over many*, *many years*, *like when he was a kid he never had any education*, *there was no*… *like they’d get like one orange every six months or something like that*, *there was just so so little*, *just so little fruit and veg”—IOT018*

“*[And]…we’re telling them to*… *we suggest*, *or we say*, *go and eat fruit and vegetables*, *but they can’t afford fruit and vegetables believe you me here*. *It’s so expensive*. *There’s also they don’t*, *a lot of people don’t actually have the self-confidence and skills in doing it”—IOT018*

Tailoring culturally appropriate interventions for different community groups (e.g. people who identify as Cocos Malay, or Chinese, or Malay, or European) was proposed by some healthcare providers as a solution to provide meaningful nutritional knowledge relevant to their eating habits. It was thought this would likely work towards empowering people to make decisions to improve their health thereby improving their management of T2D.

“*I think if we were to say three vegies and one fruit*, *that would be more achievable*. *But when you’re out on islands like that*, *the only way that I could promote five vegies was to say*, *let’s look at zapping*, *steaming in a microwave*, *frozen peas and corn with a bit of broccoli and then have raw mushroom and a raw carrot…*.*”—MA002*

However, there was a reluctance by a number of participants with experience of living in the IOT to target an individual cultural group concerned that it would suggest favouritism and reflect badly on them:

“*And I think you run into problems if you start looking like you are targeting one part of the community more than another*, *because you then disturb some of the dynamics within the Island community*. *Which is likely to ripple through to their response to the health service in general*. *And overall*, *it is a very small community*, *so if it looking like you are favouring one over another it’s going to be very apparent*.*”—IOT007*

This belief that tailoring interventions to cultural groups rather than the community as a whole could potentially upset the status quo through not fully understanding the complex social intricacies within the community impacted their willingness to provide bespoke health programs. As one participant reflected:

*I did have an idea which was sort of negated and I was told to leave it alone”—IOT017*

### Integrating the role of community and family to support proactive compliance with medical recommendations

Most participants described one of the greatest strengths of the IOT community is that it is a tight-knit community with a proactive and committed health service. In relation to attending medical and allied health appointments the community was described as:

“*a very*, *very compliant community*, *they’re very proactive with their health*, *their health is definitely a priority for them*.*”—IOT017*

The IOT Health Service performs outreach into the community by providing transport, recalls and a comprehensive chronic disease management program, which is well attended and accepted by the community.

“*I think what works quite well is we’ve got a captive audience*. *So*, *you don’t have … that are willing to come to– 99 percent of the time–willing to come up to the clinic*, *they’re happy to engage with us and they’re … they’ll come up for their regular reviews*. *So*, *I think we keep on top of that*, *I guess*, *you’d call it cycle of care*, *pretty well*”*—IOT019*

The efforts of the IOT Health Service to facilitate good medical management, have resulted in “*pretty good*” (IOT001) health outcomes for individuals with T2D (in relation to their blood glucose HBA1C levels) in the community. While the IOT Health Service has been successful with developing some empowerment around medical management for people on the island, there is still the issue of the *“real hump”* (IOT018) to overcome to address making lifestyle and behavioural changes.

Most participants believed that to achieve effective self-management of T2D healthcare professionals need to work closely with people with T2D to enable them to understand their diabetes management medically and to implement the necessary lifestyle changes.

“*Learning to engage the clients a little more*. *Addressing them as individuals and also as part of family units*. *And helping them to understand how they can self-manage their disease”—IOT007*

Participants also identified the benefits of involving family members in patients’ management of their T2D.

“*…We also try and target not just the patient but the families as well*. *So …we’ll arrange family conferences*, *if it’s a husband that comes in*, *we’ll*..*catch up with the wife as well…one of our nurses [] implemented a group exercise session targeting people with diabetes*, *and also that just gives us the opportunity to target the whole family*, *not just the one family member that’s got diabetes*.*”—IOT003*

## Discussion

The present study contributes to our understanding of providing care for people with diabetes, in particular people with T2D, in remote settings by examining the barriers and enablers of diabetes care from the perspective of those providing the care within the IOT Health Service, community leaders (for example, school principals), and administrators working and/or living in the IOT. To the best of our knowledge, this study is the first qualitative research examining barriers and enablers to diabetes care in the IOT.

The barriers and enablers identified in our present study reflect many of those found in other studies examining diabetes care in rural and remote areas: language barriers, communication, and culturally specific management measures [14–16, 25, 26]. Unlike other studies, we identified deep-rooted cultural and historic factors that have impacted and shaped the prevention and management of type 2 diabetes in the IOT. The cultural significance of food and the important role of food in maintaining social relationships is a considerable barrier to adopting a healthy diet and was observed commonly in the IOT across all communities. People with T2D often find it difficult to manage their diets at social occasions, especially those from cultural backgrounds where sharing food is highly valued [15, 16]. Self-management can be difficult for people with T2D especially if they do not feel capable or that they lack knowledge. It is important to provide support from a multidisciplinary healthcare team to combat this over time and enable people with diabetes to build a sense of empowerment and control to manage their condition [15, 16, 27].

An additional challenge, also identified in a study of people with T2D living in a remote area of Pakistan, was a lack of support from spouses and other family members, especially with extended families living together, which was identified as a barrier to making diabetes suitable food choices [15]. There is also a gendered dimension to consider in communities where women follow more traditional roles and have less control over food choices [15].

Availability of food is another key influence on dietary habits and this was clearly identified as a challenge for those in the IOT where much is sourced from outside the IOT and a reliance on less healthy food prevails. A review of the health of Pacific Islanders who also have similar diets, highlighted the need to re-establish traditional dietary habits to improve overall population health [28]. There is a need in the IOT to encourage less reliance on these imported highly processed foods, and disseminate knowledge about and awareness of what traditional foods are available and appropriate to incorporate into daily meal planning. It would also be valuable to reinforce appropriate portion size and healthy cooking methods.

Sustainability of programs and continuity of care is recognised as an ongoing challenge in remote areas where transient health workforces are common [12, 29]. Linked to this, another issue not raised in other studies, was the perception of some participants in the IOT Health Service that they were seen as outsiders with an assumption that they would not remain in the IOT long term. This may negatively impact any health outreach program or culturally tailored services the health services might adopt. Building relationships through credibility and trust in the community and having the ability to work across cultural groups is essential for developing respect and understanding. One way to build trust and meaningful relationships in the community is through having lived experience of being embedded in the community [30–32]; working with and for the community.

Our findings align with the literature that recognises the impact of language barriers between patient and provider as being significant for effective diabetes self-management in the general population, and in other culturally and linguistically diverse groups [33–35]. It is difficult to share information about the prevention and management of diabetes working through a third party interpreter, or disseminating educational materials to people who cannot read and have low health literacy. A recent review on health literacy among Aboriginal and Torres Strait Islanders found few interventions to address this have been successful and that any future efforts must involve substantial community engagement in the design and implementation of any interventions, including careful consideration of culture, and relevant languages [36]. They further highlight that such participation will only strengthen autonomy and build empowerment among the community [36].

The two enablers we identified are also supported in the literature: management, including culturally-appropriate strategies [36, 37] and family support [38]. The family unit generally is acknowledged as a great source of support for individuals with diabetes, and their involvement in part of the diabetes management plan has been shown to result in good control of diabetes [37, 38]. Therefore, a potential enabler in dietary change could be increased family support and creating change at the meso or community level, rather than at the individual level. Issues identified with communication might also explain participants’ perceptions of passive attitudes towards seeking information and difficulty obtaining and sharing detailed information about management of their diabetes.

### Implications for research policy and diabetes management practice

The barriers identified in our study have the potential to go some way to overcoming issues relating to prevention and management of type 2 diabetes, but most are untested in the IOT context. Moreover, structural barriers, such as remote settings and entrenched cultural/traditional values are difficult to address through research projects alone, they require drive and motivation from the community and *champions* to lead the change.

Interventions aimed at improving prevention and management of diabetes management in the IOT should include consumer and community members in any planning, and consider harnessing family involvement coupled with education. Importantly, any education about healthy eating needs to be culturally appropriate and tailored to the conditions and circumstances of individuals and their communities, including provision of in-language resources.

### Study strengths and limitations

A strength of this study is that it reports the findings of one of the first, as far as we are aware, qualitative studies examining barriers and enablers to diabetes care in the IOT. It fills a knowledge gap and can improve our understanding of the ways in which the IOT and other remote linguistically and culturally diverse communities can better manage and prevent diabetes. This can inform interventions to address the increasing incidence of type 2 diabetes in the IOT and potentially other rural and remote communities. However, care must be taken with regard to the transferability of this study due to the sample being drawn from one geographical location and the unique historical and cultural background of the IOT. Also, as no consumers were included as participants in the study the number of stakeholders consulted was limited. Future studies should include people with diabetes and their family members who have experience engaging with the IOT Health Services. A further limitation is that participants were not offered the opportunity to check and provide feedback on their interview transcripts.

## Conclusion

Diabetes care in the IOT faces a unique challenge due to their cultural and linguistic diversity. This study has drawn upon health care professionals and health service administrators in the IOT to identify barriers and enablers to effective type 2 diabetes care across multiple levels of influence in Australia’s most remote communities.

Many of the barriers suggested in the study, such as language barriers, communication challenges and a lack of culturally specific management measures may be applicable to both rural and remote areas. However, some of the identified barriers and enablers are unique to the IOT community, such as the historical context and the perception of health workers being seen as outsiders which may negatively impact any health outreach program or culturally tailored services the health services will adopt. Therefore, these factors need to be considered and incorporated into routine diabetes care for people with T2D to ensure successful and effective delivery of services in a remote context.

## Supporting information

S1 Table General demographics. (DOCX)

S2 Table Questions for the healthcare sector stakeholders. (DOCX)

S3 Table Questions for the education sector stakeholder. (DOCX)
