# Supplementary material for: Exploring the barriers and enablers of diabetes care in a remote Australian context: A qualitative study
Source: PLoS One. 2023 Jul 27;18(7):e0286517. doi: 10.1371/journal.pone.0286517 (PMC10373998; doi:10.1371/journal.pone.0286517)
Supplement: S1 Table — (DOCX) [file pone.0286517.s001.docx]

**Supplementary Table 1: General demographics**

| Demographics |
| --- |
| 1. Gender: □ Male □ Female |
| 2. How long have you been living on the island? |
| 3. Role: What is your role in the Indian Ocean Territories Health Service (IOTHS)? |
| 2. How long have you worked in the IOTHS? |
