# Supplementary material for: Exploring the barriers and enablers of diabetes care in a remote Australian context: A qualitative study
Source: PLoS One. 2023 Jul 27;18(7):e0286517. doi: 10.1371/journal.pone.0286517 (PMC10373998; doi:10.1371/journal.pone.0286517)
Supplement: S2 Table — (DOCX) [file pone.0286517.s002.docx]

**Supplementary Table 2: Questions for the healthcare sector stakeholders**

| Interview Questions |
| --- |
| Understanding the existing system |
| 1. We would like to understand diabetes care on the islands. How would you describe the coordination of diabetes care in the IOT?    - Prompt: GP/ Nurse/ Social workers – follow up continuity of care?    - Support provided to patients to make behaviour/ lifestyle changes    - How people diagnosed with diabetes are monitored and followed up by the health service 2. What is the attitude of people with diabetes in relation to their diagnosis?    - Is there real and sustainable behaviour change? Is there an understanding of the consequences of being a person with diabetes? 3. What are the attitudes towards healthy diet in the communities on the islands?    - Prompt: Men, Women, and children    - Cultural attribution, age, and level of education. 4. What are the attitudes towards physical activities on the islands?    - Prompt: Men, women, children.    - Community: influences, clubs, volunteering, gym activities, engagement. 5. Thinking about pregnancy and diabetes including gestational and pre-existing diabetes. Can you explain the current approach to care? (screening, management of pregnancy.)   Do they adjust well to gestational diabetes diagnosis? Prompt: adherence to lifestyle changes, efficacy in checking levels, confidence. Do you talk to them that they may be at an increased risk of developing type 2 diabetes in the future? What is their reaction – would those who change their lifestyle keep it up?   1. Are there any current programs aimed at preventing diabetes in the health service?    - Prompt: Thinking about the existing programs, who runs them (e.g. run by a GP, or by nurse or diabetes educator) or inter-institution multidisciplinary? Are these done routinely or on a case by case basis?    - What are the attitudes towards engaging in these prevention programs? 2. Do you know of any programs beyond the health system that could impact on prevention or management of diabetes (e.g. school based healthy eating programs etc.)? 3. Overall, thinking about these programs, what do you think is currently working well? 4. What do you think might be improved? |
| Understanding the current enablers on the island |
| 1. What are the characteristics on the island that you think enable good diabetes care prevention and management in the IOT?    - Prompt: If so, can you describe them to me? Attitudes of the community, Community groups/ cohesion. Health service, culture. Elements of the diet that could be encouraged more.    - Are there opportunities in the IOT community that could be harnessed to improve diabetes care or prevent diabetes on the islands? 2. What are the difficulties (for residents) in the IOT in preventing diabetes? 3. What do you think is the most important issues we need to know about of care relating to diabetes in the IOT? |
| Identification of intervention |
| 1. What kind of initiative do you think could have the most impact for managing diabetes?    - Prompt: For whom? Children, adult or elderly. How should those populations be targeted? 2. Thinking about prevention, what kind of initiative do you think could have the most impact?  - Prompt: In the area of diet and nutrition or physical activities?  1. How do we encourage participation and make programs for improving wellbeing sustainable in the IOT community?    - Rural and remote experience. Cultural etc. 2. Who else should we be talking to? Who do you think has the greatest potential influence to help fix the diabetes problems on the island? |
| How can we prioritise across different potential interventions? |
| 1. Our research aims to generate a list of possible initiatives to prevent and manage diabetes in the IOT. We are interested in your views on criteria that you think should play a role in prioritising potential initiatives for further exploration. [could potentially probe in relation to criteria often used in the literature such as health gain; equity; access for vulnerable populations; severity; cost; etc.] |
| Close |
| - Ask them if there are any other questions/ points they would like to make and if they would like a copy of the results. - Briefly summarize the information that has been recorded in this interview. - Thank them for their time |
