# Supplementary material for: Exploring the barriers and enablers of diabetes care in a remote Australian context: A qualitative study
Source: PLoS One. 2023 Jul 27;18(7):e0286517. doi: 10.1371/journal.pone.0286517 (PMC10373998; doi:10.1371/journal.pone.0286517)
Supplement: S3 Table — (DOCX) [file pone.0286517.s003.docx]

**Supplementary Table 3: Questions for the education sector stakeholder**

| Interview Questions |
| --- |
| Understanding the existing system |
| 1. Our project is about helping to prevent and manage diabetes. Do you have children with diabetes in your school? How is it managed and what is the role of teachers?    1. Type 1    2. Type 2 2. We would like to understand the current approach to Health and well-being in your school particularly around physical activities and food and drinks. Can you describe them to me? 3. Are there any current opportunities for students aimed at encouraging physical activities?    1. Active transportation to school (bike/ walking to school), extracurricular physical activity (sports, non-competitive recreation, active), facilities in school.    2. Are they organised within the school day or after school?    3. Social inclusion equal access to opportunities within physical activity and sports settings, Culturally and contextually relevant physical activity opportunities. 4. Are there any current school programs aimed at encouraging healthy diet?    1. Healthy food and drink policy, traffic light system crunch and sip.    2. Do parents pack lunch for their children?    3. Are there any restrictions on what children can bring to school to eat and drink?    4. If you so have restrictions, how well are these enforced?    5. What was the response of parents, students and staff to these rules?    6. Has there been any education provided to parents to help them create health lunchboxes? i.e. leaflets etc.? 5. Thinking about the existing programs, who runs them (e.g. teachers, other staff, community group or parent led)? 6. Overall, thinking about these programs, what do you think is currently working well? 7. What do you think might be improved? |
| Understanding the current constraints on the island |
| 1. What are the particular challenges linked to the remote location of the IOT in either encouraging physical activity and healthy diets in schools? 2. What do you think are the most important aspect to encourage physical activity and healthy eating in schools in the IOT? |
| Identification of intervention |
| 1. What kind of initiative do you think could have the most impact?    1. Prompt: For whom? Children, adult or elderly. How should those populations be targeted? 2. Thinking about prevention, what kind of initiative do you think could have the most impact?    1. Prompt: In the area of diet and nutrition or physical activities? 3. Who else should we be talking to? Who do you think has the greatest potential influence to help fix the diabetes problems on the island?    1. Prompt: Are these people outside the health service? |
| How can we prioritise across different potential interventions? |
| 1. Our research aims to generate a list of possible initiatives to prevent and manage diabetes in the IOT. We are interested in your views on criteria that you think should play a role in prioritising potential initiatives for further exploration. [could potentially probe in relation to criteria often used in the literature such as health gain; equity; access for vulnerable populations; severity; cost; etc.] |
| Close |
| - Ask them if there are any other questions/ points they would like to make and if they would like a copy of the results - Briefly summarize the information that has been recorded in this interview. - Thank them for their time |
